# Effective sympathetic nerve block for chemotherapy-induced peripheral neuropathy: a case report

**DOI:** 10.1093/omcr/omae006

**Published:** 2024-02-16

**Authors:** Aya Kuroyanagi, Chiaki Inano, Junko Adachi, Goro Kaneko, Hideki Toyokawa

**Affiliations:** Department of Anesthesiology, Saitama Cooperative Hospital, Kawaguchi, Japan; Department of Anesthesiology, Tokyo Metropolitan Police Hospital, Nakano, Japan; Department of Anesthesiology, Saitama Cooperative Hospital, Kawaguchi, Japan; Department of Anesthesiology, Saitama Cooperative Hospital, Kawaguchi, Japan; Department of Anesthesiology, Tokyo Women’s Medical University, Shinjuku, Japan

## Abstract

Chemotherapy-induced peripheral neuropathy (CIPN) is one of the most difficult-to-alleviate side effects of chemotherapy, impacting the patient’s daily activities and quality of life and frequently necessitating the discontinuation or dose reduction of anticancer drugs. An effective treatment for CIPN is yet to be established. Herein, we report the case of a patient who developed CIPN after receiving paclitaxel as postoperative chemotherapy for breast cancer. The patient experienced difficulties in performing daily activities owing to pain in her fingers and toes despite attempts to treat these symptoms with medications. Stellate and lumbar sympathetic ganglion blocks improved CIPN-induced symptoms of numbness and pain in the extremities. Thereafter, lumbar sympathetic ganglion block was performed once every 6 months, markedly improving the patient’s quality of life. Accordingly, sympathetic nerve block can facilitate pain control in patients with CIPN refractory to pharmacotherapy.

## INTRODUCTION

Chemotherapy-induced peripheral neuropathy (CIPN) is a well-known cancer chemotherapy-induced adverse event; however, an effective treatment strategy is lacking. Although several therapeutics have been explored, only duloxetine has shown moderate efficacy [1]. CIPN can interfere with the patient’s chemotherapy, daily activities, and quality of life [1, 2].

Sympathetic nerve blocks induce analgesia by suppressing sympathetic nervous system activity and also improve blood circulation in peripheral tissues [3]. However, only one case report explored the use of sympathetic nerve blocks for treating painful diabetic peripheral neuropathy [4]. We report a case of chemotherapy-induced peripheral neuropathy treated using the lumber sympathetic ganglion block (LSGB).

## CASE REPORT

**Figure 1:**
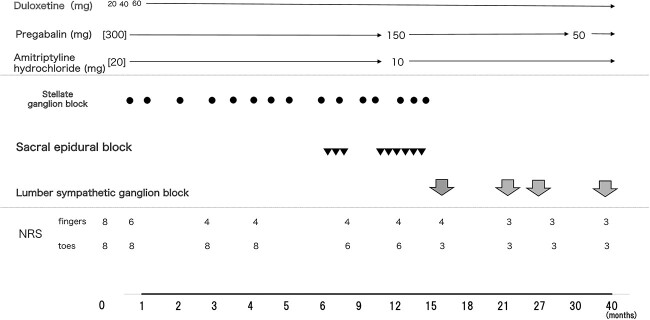
Treatment course comprising drug therapy and nerve block and changes in numerical rating scale (NRS) over 40 months from the initial visit to the end of the fourth lumbar sympathetic block. Duloxetine (20 mg) was administered at the initial visit, and the dose was increased to a maximum of 60 mg after confirming the absence of side effects. The patient’s NRS improved from 8 to 6 in both fingers, although severe pain persisted in both toes. Pregabalin was initiated at symptom onset, with the patient receiving 300 mg at the initial visit. Six months after the initial visit, the dose was reduced to 150 mg following improvement in numbness and pain in the fingers. The dose was further reduced to 50 mg owing to further improvement upon administering a sympathetic nerve block. Amitriptyline hydrochloride was initiated at 20 mg by the previous physician but was reduced to 10 mg after approximately 1 year upon observing improvements in pain and numbness in the fingers. ● Stellate ganglion block (SGB): Fifteen procedures were performed over approximately 15 months. ▼ Sacral epidural block: After improvement of upper extremity NRS, a total of nine procedures were performed over 9 months. ↓ Lumbar sympathetic ganglion block (LSGB): The first, second, third, and fourth LSGBs were performed 16, 21, 26, and 39 months after the first visit.

A 49-year-old female underwent partial mastectomy for left breast cancer, followed by paclitaxel-based chemotherapy, during which she experienced numbness and hypesthesia in the periphery of her extremities. Nonsteroidal anti-inflammatory drugs, mecobalamin, pregabalin, and amitriptyline failed to sufficiently improve symptoms. Four years after chemotherapy completion, she presented to our outpatient clinic with a chief complaint of numbness in the extremities. Initial examination revealed reduced sensations to temperature, touch, and vibration in the bilateral toes and soles. Manual muscle testing revealed that the scores of the flexor digitorum longus muscles had reduced to 4–5 points, with no marked toe motion impairment. Numbness was more severe in the toes than in the fingers. The numerical rating scale (NRS) score for both the fingers and toes was 8, and the pain DETECT questionnaire (PD-Q) score was 30. Cold stimulation aggravated pain, with severe pain necessitating absence from work. The main symptom was bilateral symmetrical numbness, with no findings suggesting peripheral neuropathy of spinal origin. Spinal magnetic resonance images showed no abnormal signals in the spinal cord. In the etiologic evaluation of neuropathy, autoimmune diseases, diabetes mellitus, or other peripheral neuropathies were also ruled out. Thus, the diagnosis of chronic CIPN with predominant sensory neuropathy due to chemotherapy drugs was established. The patient had grade 3 CIPN according to the Common Terminology Criteria for Adverse Events (CTCAE, version 5.0). In addition to the pregabalin and amitriptyline, duloxetine was initiated at 20 mg/day and subsequently increased to 60 mg/day (Fig. 1). The NRS improved to 6, although hand pain and numbness persisted. Stellate ganglion block (SGB) was performed with 1% lidocaine (5 ml), injected at the C6 level as a sympathetic nerve block. Two weeks after the initial SGB, the NRS score improved to 4, with improvement in finger numbness and pain. After 11 SGB sessions over the next 6 months, cold stimulation-induced pain and numbness improved, and the NRS score stabilized at 3. Pregabalin and amitriptyline dosages were reduced by approximately 50%. Although pain and numbness were relieved, the pain in her toes persisted; hence, a sacral epidural block was performed. After nine sacral epidural blocks, the NRS score improved from 6 to 2, with reduced numbness; however, these effects were short-lived, lasting for only a few days. Considering the potential benefit of the previous sympathetic nerve block, LSGB was performed again. After confirming the presence of the needle tip (21 G, 14.4 cm, 1 cm uninsulated tip pole needle) in the L3 and L4 bilateral sympathetic ganglia, radiofrequency thermocoagulation was performed at 90°C (180 s/site). After LSGB, the pain and numbness in the toes improved, and the NRS score improved to 2. Currently, the patient has CTCAE grade 2 neuropathy, PD-Q score of 10, and good self-reported quality of life. Since then, LSGB has been performed every 6 months, and the symptoms have remained stable. Further, as the patient’s pain gradually improved and no longer interfered with her daily activities, no psychotherapy or other interventions were necessary.

## DISCUSSION

This report discusses the case of a patient with chronic-phase CIPN, who mainly presented with neuropathy in the peripheral limbs after initiating paclitaxel. Paclitaxel reportedly damages the dorsal root ganglia in the dorsal horn of the spinal cord and causes dose-dependent sensory neuropathy [5] and mild motor neuropathy [6, 7]. In addition to peripheral nerve dysfunction, long-term changes in the central nervous system can cause chronic pain [8]. Chronic pain has been attributed to functional and morphological bridges forming between the centrifugal fibers of sympathetic nerves and nociceptive transmitting nerves, whereby sympathetic excitation is transmitted as nociception [4, 9]. Possibly, our patient developed a cross-bridge between peripheral and sympathetic nerves during disease chronicity, and the sympathetic nerve blocks (SGB and LSGB) suppressed the sympathetic nervous system activity, thereby alleviating peripheral neuropathic pain. The blockade of sympathetic centrifugal signals improved blood circulation in the peripheral tissues, which may have alleviated cold stimulation-induced pain.

In patients with sympathetically mediated leg pain, LSGB targets the lumbar sympathetic ganglia. LSGB is delivered in three methods: local anesthetic injection, nerve-destroying agents such as phenol, and high-frequency thermal coagulation. We used high-frequency thermocoagulation to thermally coagulate the nerve with high-frequency energy (70–90°C) and block nerve transmission for a prolonged period. This method is reversible, as opposed to nerve blockade with neuroleptic drugs that only block the target nerve localized at the electrode tip. In patients with complex regional pain syndrome of the lower extremities, radiofrequency thermal coagulation can be as effective as phenol-mediated nerve destruction [10]. The nerve block was not associated with any apparent side effects or complications. Although duloxetine may have contributed to analgesia, the additional LSGB-mediated improvement in leg pain suggests that sympathetic nerve block may contribute to pain relief in patients with chronic CIPN due to pharmacotherapy. In our patient, sympathetic nerve block led to good clinical results. However, since objective laboratory values were not available, the evaluation of pain and numbness after the treatment was subjective. Moreover, individual differences in the site and degree of neuropathy depending on the types and doses of anticancer drugs and duration of administration can lead to variations in the effectiveness of the nerve block among individuals; therefore, further investigations are needed. This report highlights the benefit of sympathetic nerve blocks in managing chronic CIPN.
